# Defining minimum volume thresholds to increase quality of care: a new patient-oriented approach using mixed integer programming

**DOI:** 10.1007/s10198-021-01406-w

**Published:** 2022-01-28

**Authors:** Justus F. A. Vogel, Max Barkhausen, Christoph M. Pross, Alexander Geissler

**Affiliations:** 1 School of Medicine, Chair of Health Care Management, University of St. Gallen, St. Jakob-Strasse 21, 9000 St. Gallen, Switzerland; 2 Berlin, Germany; 3 Department of Health Care Management, Berlin University of Technology, Strasse des 17. Juni 135, 10623 Berlin, Germany

**Keywords:** Mixed integer programming, Quality of care, Minimum volume thresholds, Simulation modeling, Health policy, I10, I11, I18

## Abstract

A positive relationship between treatment volume and outcome quality has been demonstrated in the literature and is thus evident for a variety of procedures. Consequently, policy makers have tried to translate this so-called volume–outcome relationship into minimum volume regulation (MVR) to increase the quality of care—yet with limited success. Until today, the effect of strict MVR application remains unclear as outcome quality gains cannot be estimated adequately and restrictions to application such as patient travel time and utilization of remaining hospital capacity are not considered sufficiently. Accordingly, when defining MVR, its effectiveness cannot be assessed. Thus, we developed a mixed integer programming model to define minimum volume thresholds balancing utility in terms of outcome quality gain and feasibility in terms of restricted patient travel time and utilization of hospital capacity. We applied our model to the German hospital sector and to four surgical procedures. Results showed that effective MVR needs a minimum volume threshold of 125 treatments for cholecystectomy, of 45 and 25 treatments for colon and rectum resection, respectively, of 32 treatments for radical prostatectomy and of 60 treatments for total knee arthroplasty. Depending on procedure type and incidence as well as the procedure’s complication rate, outcome quality gain ranged between 287 (radical prostatectomy) and 977 (colon resection) avoidable complications (11.7% and 11.9% of all complications). Ultimately, policy makers can use our model to leverage MVR’s intended benefit: concentrating treatment delivery to improve the quality of care.

## Introduction

The positive relationship between treatment volume and outcome quality as well as its theory have been analyzed by researchers for four decades [1, 2]. Empirical studies and systematic reviews discuss limitations such as the type of analyzed quality indicators (QI), methods for risk adjustment, varying data sources and patient data samples, and limited geographical scope, yet their results confirm the volume–outcome relationship across various procedures [3–8].

Thus, research focus has gradually shifted to finding the underlying reasons of the volume–outcome relationship [3, 9–12]. Moreover, studies investigate how the volume–outcome relationship can be translated into effective minimum volume regulation (MVR), in which minimum volume thresholds (MVT) are set normatively as precondition for hospitals to perform procedures and to claim reimbursement. Approaches and findings of existing studies can be summarized as follows.

- *Simulation of singular effects* Strict MVR application affects several dimensions relevant for patient care. The most commonly discussed dimensions are patient travel time, hospital capacity, the level of centralization expressed as the share of affected hospitals and patients, and the change in system level outcome quality [13–23]. So far, studies have investigated the effect of strict MVR application on only one and in some cases up to three of the above dimensions. In contrast, we designed a model that simulates the simultaneous impact of strict MVR application on all of the above dimensions.
- *Assumptions for patient choice* Once a low-volume hospital is excluded from supply due to strict MVR application, patients of this hospital must find a new hospital, i.e., they must be ‘reassigned’. The assumption made for how reassigned patients choose a new hospital dictates the simulated change in patient travel time, hospital capacity utilization and outcome quality. When reassigning patients, it is usually assumed that reassigned patients choose a new hospital based on proximity [14, 16, 17]. In contrast, we propose a model that allows patients to make a hospital choice based on maximizing outcome quality, i.e., the expected utility of receiving treatment, as well as travel time.
- *Applied methods and potential pitfalls* Generally, different statistical approaches such as the value of acceptable risk limit or risk gradient can be used to find suitable MVTs [20, 24, 25]. Nimptsch and Mansky [20], for instance, define the MVT as the number of procedures needed to achieve a complication risk below the observed average complication rate. Potential pitfalls of this and other statistical approaches are that the defined MVT remains somewhat arbitrary and that its definition is often one dimensional. For instance, it is very difficult to argue, why an MVT should be set to increase system level outcome quality to a level X (e.g., the observed average) and not Y. In addition, these approaches optimize only one dimension, outcome quality, and they are not tested towards their practical applicability. It remains unclear whether patients might be willing to choose other hospitals and potentially travel longer to receive treatment. It further remains unclear whether the remaining hospital treatment capacity after MVR application is sufficient to satisfy all treatment needs.
- *Application practice* MVR has been introduced for a range of specialized (surgical) procedures in a number of countries [26, 27]. Research investigating MVR application has shown, however, that existing MVR has not yet been applied strictly which is at least partially due to exceptions claimable by hospitals for non-compliance [26, 28–32]. Policy makers might have to weaken strict MVR application due to the dubiety of its effects [33].

With our model, we aim to clarify the effects of strict MVR application. To this end, we simulate the feasibility and estimate the utility of strict MVR application by answering the following questions.

1. Can MVR be strictly applied while retaining patient disutility in the form of patient travel time at an acceptable level?
2. After strict MVR application, is the remaining hospital capacity sufficient to provide treatment for reassigned patients?
3. If strict MVR application is feasible for a given procedure, how high is the potential system level outcome quality gain?

Our model unveils interrelations between the level of centralization, patient travel time, hospital capacity and outcome quality that have not been analyzed simultaneously before. However, understanding these interrelations is crucial to design MVR that are effective in practice. While we apply our model to the German hospital sector, its design provides a basis for similar simulations in other countries and can easily be adjusted to a different regional or political context.

## Methods

To clarify our model’s data need, we outline the used mixed integer programming model first. Second, the data input per data level (hospital vs. patient level) is described along with the used data sources.

### Model

In essence, any MVR is characterized by the definition of the regulated procedure via procedure and/or diagnosis codes, the definition of treatment types, the set MVT, the application level and exceptions claimable by hospitals [26, 27]. A description of MVR characterization and the definition of the MVR used in our model can be found in the “Appendix” (Table 2).

In our model, we simulate strict MVR application for two complex procedures (colon-/rectum resection [CRR], radical prostatectomy [RPE]), for one procedure with medium complexity (total knee arthroplasty, [TKA]), and for one procedure with relatively low complexity (cholecystectomy [CHE]). For these procedures, MVR has been issued in Germany or in other European countries [26, 27] and/or a positive volume–outcome relationship has been observed in the literature (e.g., for CHE [34]). Besides, we use QIs suitable for the medical context of the respective procedure including parts of the post-surgery, outpatient treatment phase (see Table 3 in the “Appendix”).

The mixed integer programming model works as a single objective linear optimization model subject to four constraints and is run separately for each procedure.^1^ The model’s objective is to maximize outcome quality on system level, i.e., across all reassigned patients and hospitals. Outcome quality is measured by risk-adjusted QIs for each procedure, and therefore, maximized by minimizing QI complication ratios (see Table 4 in the “Appendix” for model notation):

$$\begin{aligned} & {\text{Minimize}} \\ & C = \sum\limits_{h}^{H} {\sum\limits_{{p_{r} }}^{{P_{r} }} {A_{{p_{r} h}} \times Q_{h} } } \\ \end{aligned}$$

The term $C$ describes the system level complication ratio and ${Q}_{h}$ describes the outcome quality of hospital $h$ defined for all $h \in H$ where ${A}_{{p}_{\text{r}}h}=1$ means that reassigned patient ${p}_{\text{r}}$ is treated at hospital $h$ (${A}_{{p}_{\text{r}}h}=0$ otherwise). The following model assumptions regarding patient choice are thus inherent to the optimization operation.

- *Quality transparency* Reassigned patients know and understand the outcome quality of hospitals still supplying treatments. In addition, quality information is provided transparently, e.g., by outpatient physicians, by online public reporting platforms or by other sources.
- *Rational decision makers* Reassigned patients maximize expected utility from receiving treatment, i.e., they will choose the hospital with the highest outcome quality.

Without constraints, these assumptions would lead to an inadvertent, infeasible situation: all reassigned patients would choose the hospital with the highest outcome quality. Thus, to ensure feasibility of strict MVR application, we define constraints for both patient travel time as well as hospital capacity. Moreover, we define constraints for the exclusion of hospitals not respecting the MVR from supply and the according reassignment of affected patients:

*Subject to*

1. *Exclusion from supply* Hospital $h$ is excluded from supply if the average case volume in base year and base year-1 ${V}_{\text{h}}$ was below the set MVT $S,$
  $$\forall h \in H:O_{h} \le \frac{{V_{h} }}{S}$$
  where ${O}_{h}=1$ if *h* meets *S*, and ${O}_{\text{h}}=0$ otherwise.
2. *Patient reassignment* Patients can only be reassigned to a hospital that has not been excluded from supply and each patient can only be reassigned to exactly one hospital
  $$\forall h\in H: \sum_{{p}_{\text{r}}}^{{P}_{\text{r}}}{A}_{{p}_{\text{r}}h}\le \text{BIG M}\times {O}_{\text{h}}$$
  where BIG* M* means a large number greater than ${P}_{\text{r}},$
  $$\forall {p}_{r}\in {P}_{\text{r}}: \sum_{h}^{H}{A}_{{p}_{\text{r}}h}=1$$
3. *Patient travel time* The additional share ${t}_{\text{max}}$ of reassigned patients cannot travel more than $t$ minutes to receive treatment
  $$\forall {p}_{\text{r}}\in {P}_{\text{r}}: {B}_{{p}_{\text{r}}}\le \frac{\sum_{h}^{H}{A}_{{p}_{\text{r}}h}{T}_{{p}_{\text{r}}h}}{t}$$
  where ${B}_{{p}_{r}}=1$ if ${p}_{\text{r}}$’s travel time is above $t$ (${B}_{{p}_{\text{r}}}=0$ otherwise) and ${T}_{{p}_{r}h}$ denotes travel time of patient ${p}_{\text{r}}$ to hospital $h,$
  $$\forall {p}_{\text{r}}\in {P}_{\text{r}}: {\text{BIG} M\times B}_{{p}_{\text{r}}} \ge \left(\sum_{h}^{H}{A}_{{p}_{\text{r}}h}{T}_{{p}_{\text{r}}h}\right)-t$$
  $$\sum_{{p}_{\text{r}}}^{{P}_{\text{r}}}{B}_{{p}_{\text{r}}}\le (1+{t}_{\text{max}})\times {P}_{\text{r}t}$$
  where ${P}_{\text{r}t}$ means all reassigned patients traveling longer than $t$ (${P}_{\text{r}t}\in {P}_{\text{r}}$).
4. *Hospital capacity* Hospital $h$ cannot gain more than ${v}_{\text{max}}$ percent of ${N}_{h}$ (case volume treated at $h$ in base year),
  $$\forall h\in H:{N}_{h}^{{^{\prime}}}\le (1+{v}_{\text{max}})\times {N}_{h}$$
  where ${N}_{h}^{{^{\prime}}}$ denotes the new case volume treated at $h$ after the reassignment of patients, i.e., ${N}_{h}^{{^{\prime}}}={N}_{h}+ \sum_{{p}_{\text{r}}}^{{P}_{\text{r}}}{A}_{{p}_{\text{r}}h}$.

For a description of the used software, see the last paragraph of the data matching and cleaning section of the “Appendix”.

### Data

**Fig. 1:**
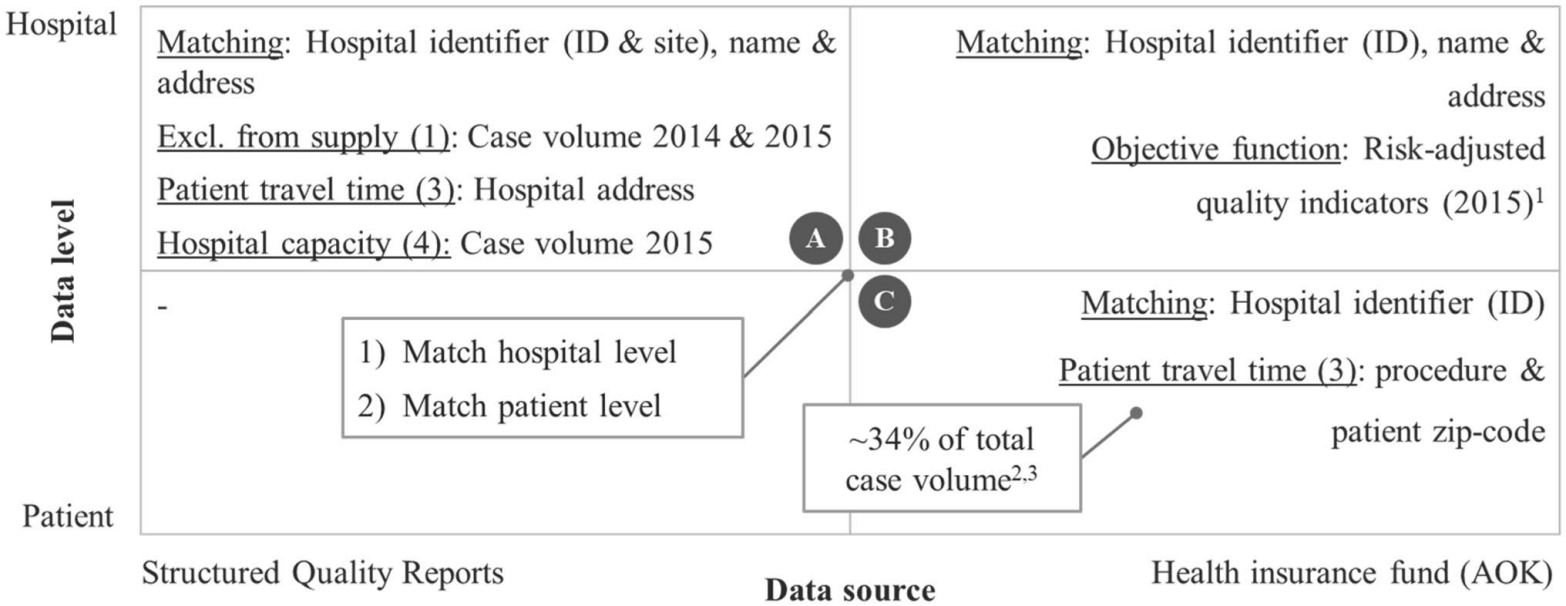
Overview of data input, levels, sources, and matching variables. Annotations: (1) If a hospital in A could not be found in B, the average quality of the respective hospital case volume quintile was assigned to that hospital. (2) Each AOK-patient represents >1 case volume. (3) If for a patient in C no matching hospital could be found in A, the patient was excluded from the sample. Moreover, if no patient could be found in C for a hospital in A, all patients from that hospital in A were assumed to live in the same zip code area as the hospital’s location

For the objective function, data from the quality assurance with routine data program from the largest German health insurance fund (*Allgemeine Ortskrankenkasse*, AOK) from 2015 were used (a recent German study provides a description of this data source [36]). If no outcome quality data were available for a hospital, the average quality of the respective hospital case volume quintile was assigned to that hospital (see Tables 5 and 6 in the “Appendix”). In addition, the model constraints use the following data input (see Fig. 1).

1. *Exclusion from supply* Case volume data from the publicly available structured quality reports of the external inpatient quality assurance program from 2015 and 2014 were used (a recent German study provides a description of this data source [37]).
2. *Patient reassignment* This constraint does not need data input.
3. *Patient travel time* Patient travel time was estimated by determining the time needed to drive with a standard passenger car from the patient zip code’s centroid to the address of the patient’s chosen hospital. The patient zip code was obtained from patient level data of the AOK. AOK-patients approximately represented 34% of total case volume ranging from 22% (RPE) to 36% (CRR). To obtain patient zip codes for each case, the non-AOK cases were equally assigned to AOK-patients. AOK-patients, therefore, received a weight greater than 1 as they represented multiple cases. If there were no patient data available for a hospital, patients were assumed to live in the hospital’s zip code area. This approximation was necessary for roughly 9% of total case volume ranging from 5% (CRR) to 13% (RPE). See Table 5 in the “Appendix” for case volume shares of all procedures.
4. *Hospital capacity* Case volume per hospital was sourced from the structured quality reports.

Data from 2015 were used for all constraints (and additionally from 2014 for the exclusion from supply constraint as indicated above). For a detailed description of the data-matching process and model computation, see the “Appendix”.

## Results

In essence, our model evaluates the tradeoff between patient disutility in terms of travel time, hospital capacity, and potential system level outcome quality gain to define effective MVTs. To unveil this tradeoff, two sets of six calculations each were conducted per procedure. Each set follows the same feasibility criteria $t$, ${t}_{\text{max}}$ and ${v}_{\text{max}}$ and only the value for the MVT $S$ is changed. This way, the marginal potential system level outcome quality gain of each MVT-increase can be compared within one calculation set. Moreover, the relationship of different feasibility criteria can be derived by comparing the two calculation sets. Besides, four sets had to be calculated for RPE as the MVR for calculation ${\text{RPE}}_{{I}_{5}}$ proved infeasible. Setting of all parameter values was derived from existing MVR in selected European countries [27], other simulations found in the literature [18] and/or loosely based on requirements from German legislation [38].

Table 1 summarizes used parameter values and the main model output per procedure and calculation and reads as follows: in the first two columns, values set for the MVT ($S$) and the travel time threshold ($t$) are shown. The third and fourth columns indicate the set and observed values for the additional share of reassigned patients that are allowed to travel longer than the travel time threshold (${t}_{\text{max}}$) and the maximum relative case volume gain per hospital (${v}_{\text{max}}$).

**Table 1:** Overview of parameter values and model output per procedure and calculation

Procedure	*I*_*n*_	*S*	*t*	*t*_max_ [set (obs.)]	*v*_max_ [set (obs.)]	Excluded hospital [abs. (rel.)]	Reassigned patients [abs. (rel.)]	Change in QI ratio [abs. (rel.)]	Avoidable complications
Cholecystectomy	*I*_1_	25	45	0.1 (0.07)	1.0 (0)	105 (8.8%)	603 (0.3%)	− 0.00 (− 0.3%)	14
	*I*_2_	50	45	0.1 (0.1)	1.0 (1)	175 (14.6%)	3291 (1.9%)	− 0.01 (− 1.2%)	58
	*I*_3_	75	45	0.1 (0.1)	1.0 (3)	270 (22.6%)	9403 (5.4%)	− 0.04 (− 4.8%)	227
	*I*_4_	100	45	0.1 (0.1)	1.0 (9)	378 (31.6%)	18,883 (10.8%)	− 0.09 (− 10.0%)	475
	*I*_5_	125	45	0.1 (0.1)	1.0 (75)	539 (45.1%)	36,885 (21.1%)	− 0.17 (− 18.1%)	861
	*I*_6_	150	45	0.1 (0.1)	1.0 (227)	676 (56.5%)	55,836 (31.9%)	− 0.21 (− 22.3%)	1061
	*I*_7_	25	30	0.1 (0.1)	1.0 (0)	105 (8.8%)	603 (0.3%)	− 0.00 (− 0.3%)	14
	*I*_8_	50	30	0.1 (0.1)	1.0 (0)	175 (14.6%)	3291 (1.9%)	− 0.01 (− 1.1%)	53
	*I*_9_	75	30	0.1 (0.1)	1.0 (2)	270 (22.6%)	9403 (5.4%)	− 0.04 (− 4.0%)	190
	*I*_10_	100	30	0.1 (0.1)	1.0 (8)	378 (31.6%)	18,883 (10.8%)	− 0.08 (− 10.0%)	389
	*I*_11_	125	30	0.1 (0.1)	1.0 (47)	539 (45.1%)	36,885 (21.1%)	− 0.17 (− 14.2%)	676
	*I*_12_	150	30	0.1 (–)	1.0 (–)	–	–	–	–
Colon resection	*I*_1_	10	45	0.1 (0.1)	1.0 (0)	149 (12.6%)	486 (0.6%)	− 0.01 (− 0.8%)	65
	*I*_2_	20	45	0.1 (0.1)	1.0 (2)	237 (20.0%)	1,711 (2.1%)	− 0.03 (− 2.5%)	205
	*I*_3_	30	45	0.1 (0.1)	1.0 (1)	324 (27.3%)	3810 (4.5%)	− 0.05 (− 5.0%)	408
	*I*_4_	40	45	0.1 (0.1)	1.0 (8)	416 (35.1%)	7049 (8.3%)	− 0.10 (− 9.2%)	760
	*I*_5_	45	45	0.1 (0.1)	1.0 (17)	467 (39.4%)	9202 (10.8%)	− 0.12 (− 11.9%)	977
	*I*_6_	50	45	0.1 (0.1)	1.0 (23)	517 (43.6%)	11,652 (13.7%)	− 0.14 (− 13.7%)	1126
	*I*_7_	10	45	0.05 (0.05)	1.0 (2)	149 (12.6%)	486 (0.6%)	− 0.01 (− 0.8%)	65
	*I*_8_	20	45	0.05 (0.05)	1.0 (3)	237 (20.0%)	1711 (2.1%)	− 0.03 (− 2.4%)	200
	*I*_9_	30	45	0.05 (0.05)	1.0 (1)	324 (27.3%)	3810 (4.5%)	− 0.05 (− 4.8%)	395
	*I*_10_	40	45	0.05 (0.05)	1.0 (10)	416 (35.1%)	7049 (8.3%)	− − 0.09 (− 8.9%)	732
	*I*_11_	45	45	0.05 (0.05)	1.0 (17)	467 (39.4%)	9202 (10.8%)	− 0.12 (− 11.4%)	939
	*I*_12_	50	45	0.05 (0.05)	1.0 (38)	517 (43.6%)	11,652 (13.7%)	− 0.13 (− 12.7%)	1048
Rectum resection	*I*_1_	5	45	0.1 (0.1)	1.25 (0)	197 (17.8%)	496 (1.5%)	− 0.01 (− 1.4%)	44
	*I*_2_	10	45	0.1 (0.1)	1.25 (3)	339 (30.7%)	1540 (4.7%)	− 0.05 (− 4.8%)	153
	*I*_3_	15	45	0.1 (0.1)	1.25 (2)	464 (42.0%)	3094 (9.4%)	− 0.10 (− 9.6%)	306
	*I*_4_	20	45	0.1 (0.1)	1.25 (8)	551 (49.8%)	4562 (13.9%)	− 0.15 (− 14.8%)	470
	*I*_5_	25	45	0.1 (0.1)	1.25 (35)	653 (59.0%)	6844 (20.9%)	− 0.20 (− 19.4%)	616
	*I*_6_	30	45	0.1 (0.1)	1.25 (81)	723 (65.4%)	8658 (26.4%)	− 0.24 (− 23.3%)	738
	*I*_7_	5	45	0.05 (0.05)	1.0 (0)	197 (17.8%)	496 (1.5%)	− 0.01 (− 1.3%)	42
	*I*_8_	10	45	0.05 (0.05)	1.0 (7)	339 (30.7%)	1540 (4.7%)	− 0.05 (− 4.6%)	146
	*I*_9_	15	45	0.05 (0.05)	1.0 (10)	464 (42.0%)	3094 (9.4%)	− 0.09 (− 9.1%)	289
	*I*_10_	20	45	0.05 (0.05)	1.0 (19)	551 (49.8%)	4562 (13.9%)	− 0.14 (− 14.0%)	445
	*I*_11_	25	45	0.05 (0.05)	1.0 (49)	653 (59.0%)	6844 (20.9%)	− 0.20 (− 17.6%)	560
	*I*_12_	30	45	0.05 (0.05)	1.0 (106)	723 (65.4%)	8658 (26.4%)	− 0.24 (− 20.5%)	650
Radical prostatectomy	*I*_1_	15	45	0.1 (0.1)	1.0 (1)	127 (30.5%)	932 (4.1%)	− 0.05 (− 4.8%)	118
	*I*_**2**_	20	45	0.1 (0.1)	1.0 (3)	155 (37.3%)	1392 (6.1%)	− 0.08 (− 7.1%)	174
	*I*_3_	27	45	0.1 (0.1)	1.0 (5)	200 (48.1%)	2420 (10.7%)	− 0.11 (− 10.1%)	249
	*I*_4_	32	45	0.1 (0.1)	1.0 (19)	225 (54.1%)	3151 (13.9%)	− 0.13 (− 11.7%)	287
	*I*_5_	35	45	0.1 (–)	1.0 (–)	–	–	–	–
	*I*_6_	35	45	0.2 (0.2)	1.0 (39)	237 (57.0%)	3553 (15.7%)	− 0.16 (− 13.9%)	342
	*I*_7_	40	45	0.2 (0.2)	1.0 (36)	259 (62.3%)	4383 (19.3%)	− 0.16 (− 14.3%)	351
	*I*_8_	45	45	0.2 (0.2)	1.0 (39)	279 (67.1%)	5190 (22.9%)	− 0.14 (− 12.2%)	301
	*I*_9_	45	45	0.3 (0.3)	1.0 (35)	279 (67.1%)	5190 (22.9%)	− 0.16 (− 13.9%)	343
	*I*_10_	35	45	0.2 (0.2)	0.8 (38)	237 (57.0%)	3553 (15.7%)	− 0.15 (− 13.1%)	321
	*I*_11_	40	45	0.2 (0.2)	0.8 (40)	259 (62.3%)	4383 (19.3%)	− 0.14 (− 12.9%)	317
	*I*_12_	45	45	0.2 (0.2)	0.8 (46)	279 (67.1%)	5190 (22.9%)	− 0.12 (− 10.6%)	261
Total knee arthroplasty	*I*_1_	20	45	0.1 (0.05)	1.0 (0)	129 (11.7%)	1196 (0.9%)	− 0.01 (− 0.9%)	40
	*I*_2_	30	45	0.1 (0.1)	1.0 (2)	222 (20.2%)	5237 (3.9%)	− 0.04 (− 4.2%)	184
	*I*_3_	40	45	0.1 (0.1)	1.0 (16)	315 (28.7%)	10,900 (8.0%)	− 0.10 (− 10.2%)	454
	*I*_4_	50	45	0.1 (0.1)	1.0 (80)	402 (36.6%)	17,196 (12.7%)	− 0.16 (− 15.4%)	681
	*I*_5_	60	45	0.1 (0.1)	1.0 (142)	527 (48.0%)	26,343 (19.4%)	− 0.20 (− 20.1%)	892
	*I*_6_	70	45	0.1 (0.1)	1.0 (145)	613 (55.8%)	26,343 (24.8%)	− 0.22 (− 22.1%)	982
	*I*_7_	20	45	0.1 (0.06)	0.8 (0)	129 (11.7%)	1196 (0.9%)	− 0.01 (− 0.9%)	40
	*I*_8_	30	45	0.1 (0.1)	0.8 (5)	222 (20.2%)	5237 (3.9%)	− 0.04 (− 4.1%)	184
	*I*_9_	40	45	0.1 (0.1)	0.8 (30)	315 (28.7%)	10,900 (8.0%)	− 0.10 (− 10.2%)	453
	*I*_10_	50	45	0.1 (0.1)	0.8 (131)	402 (36.6%)	17,196 (12.7%)	− 0.15 (− 14.8%)	658
	*I*_11_	60	45	0.1 (0.1)	0.8 (166)	527 (48.0%)	26,343 (19.4%)	− 0.19 (− 18.7%)	827
	*I*_12_	70	45	0.1 (0.1)	0.8 (176)	613 (55.8%)	26,343 (24.8%)	− 0.20 (− 20.2%)	895

Annotations: All observed absolute numbers are rounded to full values. All observed percentages are rounded to one-digit decimals. *I*_*n*_ means iteration/calculation number *n*, *S* denotes the investigated MVT, *t* denotes the travel time threshold, *t*_max_ means the additional share of reassigned patients allowed to travel longer than *t*, *v*_max_ means allowed relative case volume gain, QI means quality indicator

For calculation of ${\text{RPE}}_{{I}_{2}}$, for instance, an additional 10% of reassigned patients were allowed to travel longer than 45 min. The observed share of reassigned patients was also equal to 10%. Similarly, hospitals were allowed to gain 100% of their initial case volume at the most and three hospitals actually did gain 100% of their initial case volume. Ultimately, the observed values for ${t}_{\text{max}}$ and ${v}_{\text{max}}$ answer research questions I and II, i.e., indicate MVR feasibility. Accordingly, if observed values for these parameters are indicated with (−), the given MVR is infeasible.

If an MVR proved to be feasible, the resulting level of centralization in the form of the number and share of excluded hospitals and the number and share of reassigned patients are given in the fifth and sixth columns. Regarding ${\text{RPE}}_{{I}_{2}}$, 155 hospitals did not meet the MVT $S$ and were thus excluded from supply, representing 37.3% of the RPE hospital sample. Consequently, 1392 patients (6.1% of total) had to be reassigned.

Moreover, the resulting potential system level outcome quality gain is given first as an absolute and relative delta of the QI ratio and second as the number of avoidable complications in the last two columns. The number of avoidable complications was calculated by applying the relative outcome quality gain to the observed number of complications (see Table 3 in the “Appendix”). With respect to ${\text{RPE}}_{{I}_{2}}$, the complication ratio was 0.08 points or 7.1% lower after strict MVR application representing 174 avoidable complications, i.e., reoperations in the case of RPE. In essence, these last two columns answer research question 3, i.e., determine the potential system level outcome quality gain of feasible MVR. For a detailed overview of all model outputs, see Table 7 in the “Appendix”.

**Fig. 2:**
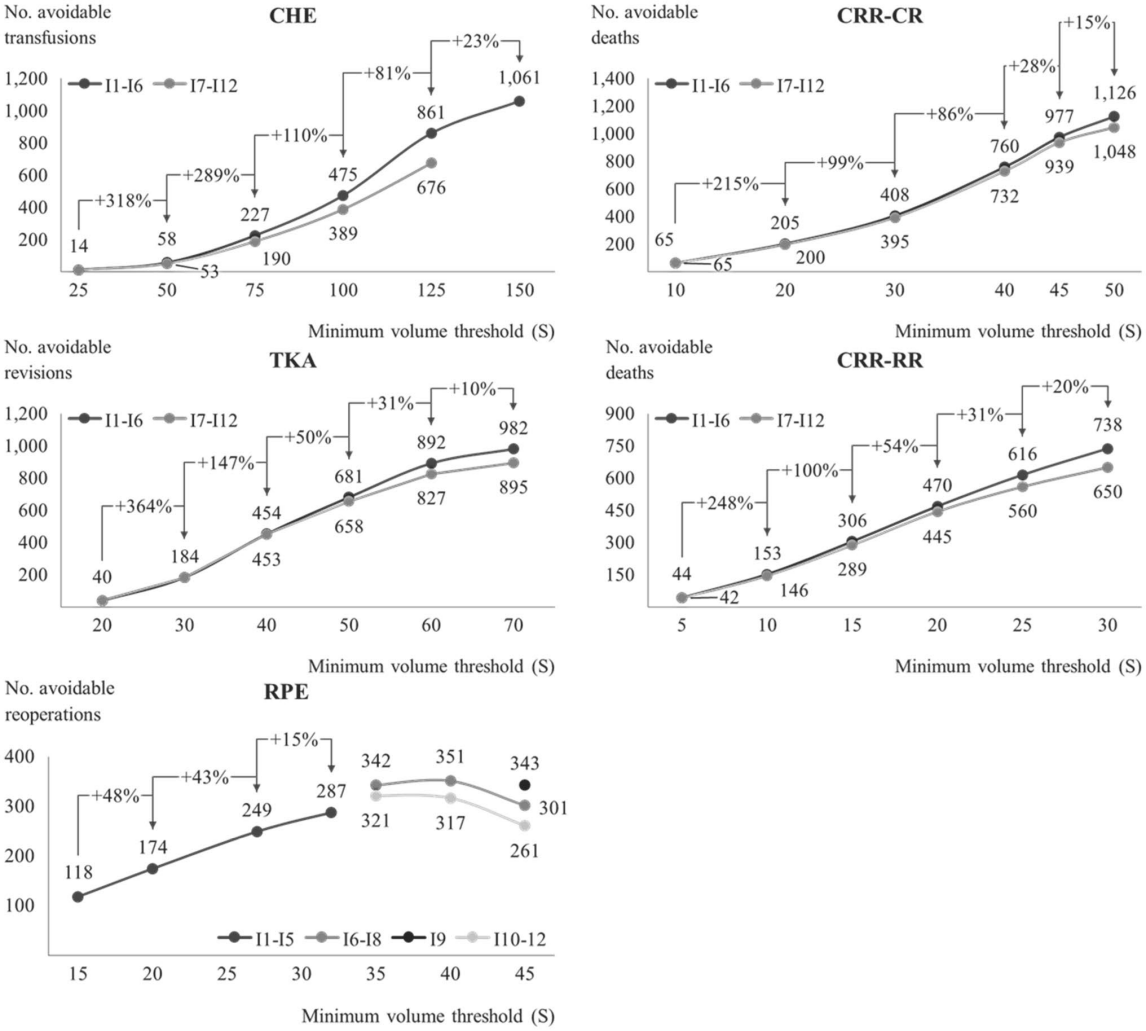
Relationship between system level outcome quality gain and MVT per procedure

To be effective, an MVT needs to balance feasibility and patient disutility with potential system level outcome quality gains. To this end, Fig. 2 visualizes relationships between marginal system level outcome quality gains (*y*-axis) and set MVTs (*x*-axis). For all procedures except RPE, the first three calculations I_1_, I_2_ and I_3_ yield high relative marginal outcome quality gains between 99 (${\text{CR}}_{{I}_{3}}$) and 364% (${\text{TKA}}_{{I}_{1}}$). For the fourth and fifth calculations, the slope starts to flatten. Moreover, disutility reaches a high level and realizing feasibility is more difficult (see Table 1 and Table 7 in the “Appendix”). Accordingly, the average absolute case volume gain per hospital becomes rather large and the additional number of minutes reassigned patients have to travel increases strongly (see Table 7 in the “Appendix”). Thus, in the case of CR, the MVT for *I*_5_ and *I*_6_ was increased less strongly than for previous calculations. Overall, MVT increases for calculations *I*_5_ and *I*_6_ are linked to higher patient disutility, more difficult realization of feasibility, and smaller marginal utility increases. Consequently, it can be deduced that the most effective MVR for all procedures except RPE requires an MVT close to *I*_5_.

The lighter gray line depicts the second calculation set (*I*_7_–*I*_12_) for which at least one feasibility constraint per procedure was set more strictly. For all procedures, the potential system level outcome quality gain is only slightly lower for the second calculation set, especially for the first four calculations. This is another indication that the MVT should not be increased further than *I*_5_. Besides, as the second calculation set yields significant system level outcome quality gains with stricter feasibility criteria, it can be deduced that strict MVR application can create high patient utility even when feasibility is more constraint.

With respect to RPE, the MVR tested in *I*_5_ proved infeasible (see Table 1). Therefore, feasibility criteria had to be relaxed for *I*_6_–*I*_8_, increasing patient disutility and making feasibility more difficult (see Table 7 in the “Appendix”). Counterintuitively, the potential system level outcome quality gain for *I*_8_ was lower than for previous calculations of the same calculation set. The same occurred for *I*_12_. This negative relationship starting after an MVT of $S=40$ is likely to be due to the distribution of QI values across our hospital sample (see Table 6 in the “Appendix”) and the limited availability of outcome quality data (see “Discussion”). Overall, an MVT close to 32 treatments (*I*_4_) seems to be most effective for RPE as patient disutility is higher and the realization of feasibility is more difficult for higher MVTs.

## Discussion

The results of our model show that strict MVR application can effectively increase quality of care while controlling for patient travel time increases and the availability of hospital capacity. The flexibility of the model allows for the integration of different quality measures, the application to different procedures as well as the consideration of different geographical, political and infrastructural circumstances. Our model can, therefore, be used in diverse settings and different countries supporting health policy makers to define effective MVTs based on empirical evidence.

To put our results in perspective to prior research, we discuss assumptions and results regarding patient travel time, hospital capacity, and outcome quality. Lastly, limitations are discussed and concluding remarks are given.

### Patient travel time

A common assumption is that reassigned patients base their hospital choice on proximity rather than outcome quality. Two previous studies on strict MVR application [16, 17], for instance, compare patient travel time changes by estimating the average travel time of both all and only reassigned patients had they chosen the closest hospital to their home, respectively, the next closest hospital providing care.

In contrast, we assume that reassigned patients do not maximize their utility solely by minimizing incurred patient travel time. In our model, patients balance utility increases due to higher quality treatment and disutility increases due to longer travel times. Patient travel time is considered as a constraint: most patients (equal to ${P}_{\text{r}t}(1-{t}_{\text{max}})$) take the disutility of hospital choice into account yet only if it exceeds a certain level ($t$) while some patients do not value travel time at all (equal to ${P}_{\text{r}t}\times {t}_{\text{max}}$). This design of the expected utility function of patients was chosen as it is in line with empirical evidence concerning patients’ preferences [23, 39–44]. Besides, the authors of previous studies also acknowledge that their data show that a considerable share of patients initially do not choose the closest hospital [16, 17]. We found this fact confirmed in our patient level data (6–16% of reassigned patients initially travel longer than $t$, see Table 7 in the “Appendix”).

Regarding results from patient travel time simulations, one study also investigates the procedure total knee arthroplasty, among others [17]. Not considering the above-discussed difference of the authors’ travel time approach compared to our approach, patient travel time increases were moderate after strict MVR application. Accessibility was thus not restricted by the investigated MVT equal to 50. Both of these findings are in line with our results.

### Hospital capacity

The use of case volume might seem unjustified and the level of allowed case volume gain unrealistic. Supporting arguments for our approach are as follows.

- *Relationship between case volume and hospital beds* Using the average length of stay, hospital beds required for additional case volume can be calculated. For ${\text{RPE}}_{{I}_{2}}$, for instance, average case volume gain per hospital amounts to 13 cases (see Table 7 in the “Appendix”). Assuming an average length of stay for a patient undergoing RPE, e.g., to treat prostate carcinoma, of 9.14 days^2^ and 80% bed utilization, hospitals on average need an additional bed capacity of 0.42 beds per year to treat 13 additional RPE cases. Presuming an average hospital ward size of 35 beds, this amounts to an additional relative capacity need of 1.2%.
- *Hospital specialization* It can be expected that to gain cases of complex procedures and to strengthen specialization, hospitals will forgo treatment of less complex procedures. After strict MVR application and patient reassignment, these hospitals might, therefore, clear capacity to treat more cases of the regulated, complex procedure.

### Outcome quality

The number of avoidable complications per procedure might seem rather high. The interpretation of the simulation results should be clear, however: due to the assumptions for patient choice, the number of avoidable complications represent the *maximum* number of complications that can be avoided by strict MVR application, hence the term *potential* system level outcome quality gain. In this context, our results show that strict MVR application with MVTs of calculation I_5_ can potentially reduce complications between 11.9 (colon resection) and 20.1% (total knee arthroplasty). Moreover, feasibility in terms of patient travel time and hospital capacity was given for 58 out of 60 calculations. Therefore, it can be concluded that strict MVR application is feasible and can yield high-outcome quality gains.

In comparison, the authors of a previous study derived system level outcome quality changes in terms of population impact numbers, i.e., the number of patients for which one complication could have been avoided [20]. For the observed period from 2009 to 2014, MVTs for the procedures colorectal resection for carcinoma and for the procedure colorectal resection for diverticulosis were set to 82 and 44. Strict application of these MVTs would yield one in 197, respectively, one in 364 avoidable deaths. Linking these population impact numbers with the total case volumes of 331,000 and 179,000, for the procedure colorectal resection for carcinoma 280 lives and for the procedure colorectal resection for diverticulosis 82 lives per year could have been saved.

These two procedures together resemble more or less the procedure CRR investigated in this study. However, outcome quality changes for only one MVT were estimated, six data years were investigated jointly and a different QI (inpatient mortality) not considering inadvertent events after hospital discharge was used. For these reasons, the affected patient samples differ between the authors’ study and our study. Moreover, the authors did not test MVR feasibility but implicitly assumed that there would be no conflicts in terms of patient travel time and hospital capacity. Thus, the question of feasibility and (political) practicability remains and, not surprisingly, the used MVTs are rather high compared to the MVTs used in our study.

### Limitations

Our approach is limited by data availability and model assumptions. With respect to patient travel time, patient level data were available for merely a subset of cases. Thus, to be able to calculate patient travel time for all cases, patients were reassigned in groups. In addition, for some reassigned patients per calculation, no observed patient zip codes were available and these patients’ hospital zip codes had to be used. As no complete set of patient level data is available to research in Germany, this approach had to be chosen. In addition, as centroids of zip codes were used and as the share of patients that zip codes were available for per procedure was comparatively high (see Table 4 in the “Appendix”), this approach should deliver acceptable results. Still, we might underestimate the actual average travel time per reassigned patient before strict MVR application (see Table 7 in the “Appendix”), as a certain number of reassigned patients is assumed to live very close to the treating hospital. Regarding our patient travel time constraint, this is a rather prudent assumption, however, as the constraint compares the number of reassigned patients traveling longer than $t$ before and after strict MVR application and all patients that are assumed to live in the same zip code area as their respective hospital’s location naturally do not travel longer than $t.$

Regarding outcome quality, the quality rating per hospital and procedure should ideally be based on several QI values and multiple data years. This way, outcome quality could be captured holistically and statistical chance would be reduced. Due to the limited data available for this study, outcome quality is based on merely one QI per procedure reported in 1 year, however.

Concerning model assumptions, a high degree of quality transparency was assumed which indubitably is an ideal state. Still, studies suggest that the degree of quality transparency is increasing [36, 37, 43]. In Germany, this is at least partially due to various quality transparency initiatives such as the external inpatient quality assurance program, the so-called White List (*Weisse Liste*) online platform and the quality assurance with routine data program brought forth by the AOK, to name just a few. In addition, it can be argued that outpatient physicians treating patients prior to their inpatient treatment recommend hospitals with higher outcome quality [45], adding to the degree of quality transparency. Besides, the quality transparency assumption was made deliberately as model results are to show the maximum outcome quality gain that can possibly be attained.

Apart from outcome quality, other parameters relevant for patients’ hospital choice such as patient travel time, structural quality indicators, general hospital characteristics (e.g., number of beds), waiting time, other outcome quality indicators (e.g., patient reported), service quality, certifications, etc. could be added to the objective function. Gutacker et al. [46] and Kuklinski et al. [47] evaluate the influence of such parameters on patients’ hospital choice. Their work could serve as a basis for developing an objective function apt to simulate patients’ decision processes more realistically. This way, the change in outcome quality for a certain MVT could be assessed more precisely.

Moreover, immediate exclusion in case of MVR non-compliance was assumed. The authors of a previous simulation study, on the other hand, employ two different scenarios [17]: immediate and successive exclusion from supply. The successive exclusion scenario assumes that first the group of hospitals with the lowest number of treatments is excluded and patients of those hospitals are reassigned, subsequently the group with the second lowest and so forth. This approach gives hospitals that initially do not comply with the MVR a chance to gain enough case volume from reassigned patients to eventually comply with the MVR. Consequentially, those hospitals are not excluded from supply even though they would have been excluded in the immediate exclusion scenario. MVR in Germany issued by the Joint Federal Commission does grant a 2-year transition period in case of the introduction of MVR [38] for a new procedure which might justify a successive exclusion scenario. Evidence suggests, however, that during a transition period, only a relatively small share of hospitals “voluntarily” refrains from performing treatments [28, 30]. Therefore, the immediate closure scenario was chosen.

## Conclusion

There are studies simulating the effect of strict MVR application on the centralization of hospital services, outcome quality and/or patient travel time. No study exists that simultaneously simulates the effect of strict MVR application on all dimensions relevant for patient care, however. On the one hand, patient travel time and hospital capacity need to be considered to ensure feasibility and (political) practicability of strict MVR application. The degree of potential system level outcome quality gain, on the other hand, represents the utility of strict MVR application. Effective MVR balance feasibility and potential outcome quality gain.

Our model objectifies the discussion concerning the definition of MVTs. Health policy makers can use our model to demonstrate first that strict application for a certain MVR is feasible and second how strongly it can increase the quality of care. Health insurance funds as well as hospital networks can use our model to evaluate how MVR is likely to affect their insurees or hospital sites. Lastly, with our model results, patients are able to comprehend tradeoffs associated with MVR.

For Germany, we demonstrated that effective MVR can be designed and should thus be introduced (CHE, CRR, RPE), respectively, adapted (TKA) and strictly applied for all investigated procedures. The same can be tested for any procedure and country given the availability of hospital and patient level data. We deliver our model and results at an opportune moment to enable health policy makers to leverage MVR’s intended benefit: concentrating care at high-quality centers to improve the quality of care.

## Data Availability

Detailed model results are available upon request. Raw data cannot be shared due to confidentiality.

## Appendix

### Characteristics of MVR

In essence, an MVR for a given procedure can be described by the following five characteristics [26, 27, 38].

1. *Definition of procedure* Procedure codes are most commonly used to determine the scope of a procedure. In Germany, codes from the Operation and Procedure Catalogue (*Operationen- und Prozedurenschlüssel, *OPS) are used, for instance. Some organizations additionally use diagnosis codes, e.g., from the International Classification of Diseases (ICD), for their definition. Even if the names of procedures are similar, the codes used procedure definition might differ in number and level of detail, changing the effect of the MVR by enlarging or reducing the affected patient group. In our model, we define procedures solely by procedure codes.
2. *Definition of treatment types within a procedure* Procedures always consist of at least one treatment type and in many cases of more than one treatment type. Each treatment type is in turn defined by a subset of (procedure) codes that were used for the procedure definition. The MVR’s effect is only amplified, however, if separate thresholds are defined for each treatment type (see next characteristic). In our model, two treatment types are defined for the procedure CRR, namely Colon Resection (CR) and Rectum Resection (RR).
3. *Threshold* The MVT is an essential part of an MVR. Still, an MVT is not always defined, e.g., for the procedure *coronary surgery* in Germany. The MVT has significant impact on the effect of any given MVR which is amplified if MVTs are defined separately for each treatment type as then multiple requirements have to be met for MVR compliance. In our model, we test multiple MVTs for each procedure. Moreover, separate MVTs are defined and modeled for CR and RR.
4. *Application level* An MVR can define MVTs for either each treating physician and/or for the hospital as a whole, i.e., the sum of all treatments of all treating physicians. In our model, MVR is always applied on hospital level.
5. *Exceptions* Organizations can define exceptions for hospitals not meeting an MVR. Examples of exceptions claimable in Germany include structural or staff-related changes, the (re-)opening of a certain specialty and/or transition periods when introducing MVR for new procedure or on surgeon level. When MVR is applied strictly as in our model, no exceptions are granted.

In practice, the specification of the above characteristics depends on the issuing organizations’ differing regulation goals and methods. The Joint Federal Committee (*Gemeinsamer Bundesausschuss*) in Germany, an example for a legislative organization, uses MVR to prevent hospitals from opportunistic, occasional (surgical) treatment via withholding reimbursement for these treatments in cooperation with health insurance funds (Table 2).

**Table 2:** Characterization of MVR per procedure used for modeling

Procedure	Def. of procedure	Def. of treatment type	MVT	Application level	Exceptions	Organization (i.e., source)
CRR	5–455, 5–4565-484, 5–485, 5–486.3, .4	CR: 5–455, 5–456 RR: 5–484, 5–485, 5–486.3, .4	According to calculation	Hospital	None	German society for general and visceral surgery
CHE	5–511.0 to .3,.x,.y	None				Scientific Institute of the AOK
RPE	5–604					Own definition
TKA	5–822.9, .g, .h, .j, .k					G-BA

Annotation: Only the highest level of procedure codes is shown

### Quality indicator selection (Table 3)

**Table 3:** Overview of quality indicators and observed rate of complications per procedure

Procedure	Risk-adjusted QI	Observed complication rate/no. of complications
CHE	7-day blood transfusion	2.7%/4728
CRR	90-day mortality	9.7% (CRR)/8231 (CR); 3175 (RR)
RPE	1-year reoperation	10.8%/2461
TKA	1-year revision	3.3%/4435

Annotation: QIs were chosen based on the observed volume–outcome relationship in the raw data and the number of observed complications

Source: QI data from the quality assurance with routine data program, provided by the Scientific Institute of the AOK from 2015

#### Model notation (Table 4)

**Table 4:** Model notation

Parameter	Description
*C*	Risk-adjusted complication ratio on system level
${Q}_{\text{h}}$	Risk-adjusted outcome quality of *h* defined over all $h \in H$
*H*	Set of hospitals
*P*	Set of all patients
${P}_{\text{r}}$	Set of reassigned patients
${P}_{\text{r}t}$	Set of reassigned patients that travel longer than $t$ in base year
*S*	Minimum number of required treatments (i.e., MVT)
${V}_{\text{h}}$	Average case volume treated at *h* of base year and base year-1 (compared with *S*)
${O}_{\text{h}}$	Decision variable defined over all $h \in H$. Given the defined constraints, ${O}_{h}$ = 1 if *h* meets *S*, ${O}_{h}$ = 0 otherwise; $${O}_{h}\in \left\{\begin{array}{c}1\\ 0\end{array}\right.$$
${A}_{{p}_{\text{r}}h}$	Decision variable defined over all $p\in P, h \in H$. Given the defined constraints, the intuitive meaning of ${A}_{{p}_{r}h}$ = 1 is that patient ${p}_{r}$ is treated at hospital $h$, ${A}_{{p}_{r}h}$ = 0 otherwise; $${A}_{ph}\in \left\{\begin{array}{c}1\\ 0\end{array}\right.$$
BIG *M* > $P$	A large number greater than *P*
${T}_{{p}_{\text{r}}h}$	Travel time of patient ${p}_{\text{r}}$ to hospital $h$, defined over all ${p}_{\text{r}}\in {P}_{\text{r}}$, $h \in H$
$t$	Normatively set travel time threshold
${t}_{\text{max}}$	Maximum additional share of reassigned patients allowed to travel longer than $t$
${B}_{{p}_{\text{r}}}$	Defined over all ${p}_{\text{r}}\in {P}_{\text{r}}$, Given the defined constraints, ${B}_{{p}_{\text{r}}}=1$ if ${p}_{r}$’s travel time is above $t$, ${B}_{{p}_{\text{r}}}=0$ otherwise; $${B}_{{p}_{\text{r}}}\in \left\{\begin{array}{c}1\\ 0\end{array}\right.$$
${v}_{\text{max}}$	Maximum increase in case volume for hospital $h$
${N}_{h}$	Case volume treated at $h$ in base year
${N}_{h}^{{^{\prime}}}$	New case volume treated at $h$ after reassignment of patients

### Data matching and cleaning

As can be seen in Fig. 1 in “Methods”, we used three data sources to obtain all necessary data for modeling. To create valid data input, these three data sources were matched. To this end, the structured quality report hospital level data (A) served as the master data source, i.e., all hospital (B) and patient level data (C) were matched with A. In each data source, hospitals could be identified with a unique hospital identifier that describes one hospital, respectively, hospital group. In A, also single hospitals, i.e., hospital sites, could be identified as site numbers were given. As MVR in Germany is applied to hospital sites and not hospitals or hospital groups, it is vital to match data on hospital site level. With the term “hospital”, we, therefore, always mean a single hospital site. Suitably, using the hospital site addresses and hospital names as additional matching variables, B could be matched with A on hospital site level. In a second step, as B and C were supplied by the same data source and hospital identifiers were the same, patients in C could be matched to hospitals in B and thus to A.

As the model was designed to simulate the effect of strict MVR application on the entire German hospital sector, no hospitals in A could be excluded from the sample. Thus, missing data points had to be estimated if for a hospital in A no information could be found in either B or C (see Table 5 below and Fig. 1 in the main text).

- *Hospital in A cannot be found in B* To make our model operational, each hospital needs a QI for each procedure. In some cases (17% of hospitals respectively 10% of cases overall, ranging from 16 to 18%, respectively, 8–15% per procedure), a hospital in A could not be found in B. In this case, the average value of the QI of the hospital’s respective case volume quintile was assigned to that hospital. In a first step, total case volume of a procedure was divided in five equal parts (i.e., quintiles). In a second step, hospitals were ranked according to their case volume, smallest to largest. In a third step, the first case volume quintile was “filled” with the smallest case volume hospitals until the needed total case volume of the first quintile was reached. This was repeated for each quintile and for each procedure. For the distribution of hospitals per quintile and procedure, see Table 5 below.
- *Hospital in A cannot be found in C* To calculate patient travel time, each patient’s location, i.e., the geographical center of the patient’s zip code, is needed. If for the case volume of a hospital in A, no patient reference could be found in C, all patients of that hospital in A were assumed to live in the same zip code as the hospital’s location. This occurred for 9% of the overall case volume, ranging from 5 to 13% per procedure.

**Table 5:** Overview of data matching results

Share of…	CHE (%)	CRR (%)	RPE (%)	TKA (%)	Total (%)
AOK patients share of case volume	34	36	22	35	34
Hospitals in A not in B	17	18	16	17	17
*… according case volume share*	10	8	15	12	10
Hospitals in A not in C	15	14	13	16	15
*… according case volume share*	9	5	13	12	9
Hospitals in B not in A	1	1	< 1	1	1
*… according case volume share*	< 1	< 1	< 1	< 1	< 1
Patients in C without hospital in A	1	1	< 1	1	1

Annotation: Percentages are rounded

Data year: 2015

Lastly, in two cases, data were excluded from modeling (see Table 4).

- *Hospital in B could not be found in A* QI values of these hospitals were disregarded as they could not be matched with case volume data in A. As case volume and hospital data in A were unaffected by this, model validity was not harmed by the exclusion of these data. Besides, this occurred for only 1% of hospitals in B.
- *Patients in C that could not be assigned to a hospital in A* These patients were excluded from modeling without deteriorating model validity as this did not affect overall case volume in A. Incidentally, this occurred for only 1% of patients in C.

The model algorithm was coded and run in a Python environment which was operated on a Linux terminal. Zip codes were geocoded using Google Maps APIs and average travel times were calculated using the OpenMaps tool backend with a local Open Source Routing Machine server. For the optimization problem, the Gurobi solver was used (Table 6).

**Table 6:** Overview of case volume quintiles

Procedure	Very low	Low	Medium	High	Very high
CHE					
Total case volume	34,956	34,852	34,916	34,999	35,104
No. of hospitals	529	240	183	143	101
Median case volume (IQR)	72 (33–101)	144 (136–154)	190 (179–202)	243 (230–261)	319 (298–367)
Average value of QI	0.92	1.06	0.90	0.98	0.85
No. (share) of hospitals without QI	152 (29%)	26 (11%)	17 (9%)	3 (2%)	8 (8%)
CRR-CR					
Total case volume	16,975	16,988	17,022	16,846	17,159
No. of hospitals	621	224	157	112	72
Median case volume (IQR)	27 (8–45)	75 (68–85)	107 (98–117)	150 (138–161)	212 (194–258)
Average value of QI	1.15	1.15	0.92	1.00	1.02
No. (share) of hospitals without QI	201 (32%)	13 (6%)	8 (5%)	3 (3%)	2 (3%)
CRR-RR					
Total case volume	6552	6545	6544	6566	6579
No. of hospitals	650	200	126	82	48
Median case volume (IQR)	9 (3–16)	32 (28–37)	51 (47–56)	90 (75–114)	124 (106–142)
Average value of QI	1.15	1.07	0.97	0.99	0.96
No. (share) of hospitals without QI	142 (22%)	12 (6%)	2 (2%)	8 (10%)	4 (8%)
RPE					
Total case volume	4532	4498	4511	4291	4856
No. of hospitals	265	82	44	18	7
Median case volume (IQR)	16 (7–26)	52 (47–64)	100 (85–117)	228 (167–308)	486 (428–819)
Average value of QI	1.27	1.16	1.25	0.97	0.96
No. (share) of hospitals without QI	55 (21%)	6 (7%)	3 (7%)	1 (6%)	2 (29%)
TKA					
Total case volume	27,115	27,139	27,064	27,121	27,415
No. of hospitals	567	240	148	94	49
Median case volume (IQR)	53 (28–66)	113 (100–126)	183 (163–200)	281 (253–323)	492 (437–613)
Average value of QI	0.92	1.06	0.90	0.98	0.85
No. (share) of hospitals without QI	122 (22%)	31 (13%)	16 (11%)	16 (17%)	3 (6%)

Annotation: All absolute numbers and percentages are rounded to full values. Values for QIs are rounded to two digits

Data year: 2015

### Detailed model results (Table 7)

**Table 7:** Overview of changes for all model outputs

Procedure	*I*_*n*_	Stage	*P*_r_ traveling longer than *t*		Average patient travel time		Average case volume gain per hospital		System level outcome quality [abs. (rel.)]	
			Abs	Rel. to *P*_r_	*P*	*P*_r_	Abs	Rel	Average	Stand. Dev.
*Cholecystectomy*	*I*_1_	Base year	34	5.7%	11	20	–	–	0.94	0.99
		After MVR	78	12.9%	11	49	3	3.2%	0.94	0.99
		∆ MVR-BY	44	7.3%	0	29	–	–	0.00 (− 0.3%)	− 0.01 (− 0.6%)
	*I*_2_	Base year	219	6.7%	11	10	–	–	0.94	0.99
		After MVR	548	16.6%	11	28	13	12.5%	0.93	0.95
		∆ MVR-BY	329	10.0%	0	17	–	–	− 0.01 (− 1.2%)	− 0.04 (− 4.2%)
	*I*_3_	Base year	672	7.1%	11	10	–	–	0.94	0.99
		After MVR	1613	17.1%	12	34	36	21.9%	0.89	0.93
		∆ MVR-BY	940	10.0%	1	24	–	–	− 0.04 (− 4.8%)	− 0.06 (− 6.2%)
	*I*_4_	Base year	2076	11.0%	11	15	–	–	0.94	0.99
		After MVR	3964	21.0%	13	35	72	38.8%	0.84	0.92
		∆ MVR-BY	1888	10.0%	2	20	–	–	− 0.09 (− 10.0%)	− 0.08 (− 7.7%)
	*I*_5_	Base year	4251	11.5%	11	14	–	–	0.94	0.99
		After MVR	7939	21.5%	12	20	126	68.1%	0.77	0.87
		∆ MVR-BY	3688	10.0%	1	6	–	–	− 0.17 (− 18.1%)	− 0.12 (− 11.8%)
	*I*_6_	Base year	6483	11.6%	11	14	–	–	0.94	0.99
		After MVR	12,067	21.6%	13	20	180	84.1%	0.73	0.82
		∆ MVR-BY	5584	10.0%	2	6	–	–	− 0.21 (− 22.3%)	− 0.18 (− 17.8%)
	*I*_7_	Base year	73	12.1%	11	20	–	–	0.94	0.99
		After MVR	133	22.1%	11	38	3	3.3%	0.94	0.99
		∆ MVR-BY	60	10.0%	0	18	–	–	0.00 (− 0.3%)	− 0.01 (− 0.6%)
	*I*_8_	Base year	381	11.6%	11	10	–	–	0.94	0.99
		After MVR	710	21.6%	11	17	11	9.4%	0.93	0.95
		∆ MVR-BY	329	10.0%	0	7	–	–	− 0.01 (− 1.1%)	− 0.04 (− 4.3%)
	*I*_9_	Base year	1041	11.1%	11	11	–	–	0.94	0.99
		After MVR	1981	21.1%	12	32	30	20.8%	0.90	0.93
		∆ MVR-BY	940	10.0%	1	22	–	–	− 0.04 (− 4.0%)	− 0.07 (− 6.6%)
	*I*_10_	Base year	2846	15.1%	11	15	–	–	0.94	0.99
		After MVR	4734	25.1%	11	18	46	27.4%	0.86	0.91
		∆ MVR-BY	1888	10.0%	0	3	–	–	− 0.08 (− 8.2%)	− 0.08 (− 8.4%)
	*I*_11_	Base year	5855	15.9%	11	15	–	–	0.94	0.99
		After MVR	9544	25.9%	12	18	88	46.6%	0.80	0.87
		∆ MVR-BY	3688	10.0%	1	3	–	–	− 0.13 (− 14.4%)	− 0.12 (− 12.4%)
	*I*_12_	Base year	–	–	–	–	–	–	–	–
		After MVR	–	–	–	–	–	–	–	–
		∆ MVR-BY	–	–	–	–	–	–	–	–
*Colon resection*	*I*_1_	Base year	48	9.9%	13	15	–	–	1.04	0.87
		After MVR	96	19.8%	13	44	4	7.9%	1.03	0.85
		∆ MVR-BY	48	10.0%	0	29	–	–	− 0.01 (− 0.8%)	− 0.02 (− 2.8%)
	*I*_2_	Base year	195	11.0%	13	15	–	–	1.04	0.87
		After MVR	372	21.0%	14	56	9	18.2%	1.01	0.83
		∆ MVR-BY	177	10.0%	1	40	–	–	− 0.03 (− 2.5%)	− 0.04 (− 4.8%)
	*I*_3_	Base year	359	9.4%	13	15	–	–	1.04	0.87
		After MVR	740	19.4%	14	50	19	24.9%	0.99	0.80
		∆ MVR-BY	381	10.0%	2	35	–	–	− 0.05 (− 5.0%)	− 0.07 (− 7.6%)
	*I*_4_	Base year	714	10.1%	13	16	–	–	1.04	0.87
		After MVR	1419	20.1%	15	42	36	44.3%	0.94	0.77
		∆ MVR-BY	705	10.0%	2	26	–	–	− 0.10 (− 9.2%)	− 0.10 (− 11.2%)
	*I*_5_	Base year	930	10.1%	13	15	–	–	1.04	0.87
		After MVR	1850	20.1%	16	46	47	51.9%	0.92	0.75
		∆ MVR-BY	920	10.0%	3	31	–	–	− 0.12 (− 11.9%)	− 0.12 (− 13.7%)
	*I*_6_	Base year	1203	10.3%	13	16	–	–	1.04	0.87
		After MVR	2369	20.3%	17	45	58	59.3%	0.90	0.75
		∆ MVR-BY	1165	10.0%	4	29	–	–	− 0.14 (− 13.7%)	− 0.12 (− 14.0%)
	*I*_7_	Base year	48	9.9%	13	16	–	–	1.04	0.87
		After MVR	72	14.8%	13	57	3	9.1%	1.03	0.85
		∆ MVR-BY	24	4.9%	0	41	–	–	− 0.01 (− 0.8%)	− 0.02 (− 2.8%)
	*I*_8_	Base year	195	11.0%	13	16	–	–	1.04	0.87
		After MVR	283	16.0%	13	40	8	17.1%	1.01	0.83
		∆ MVR-BY	88	5.0%	0	24	–	–	− 0.03 (− 2.4%)	− 0.04 (− 4.8%)
	*I*_9_	Base year	359	9.4%	13	16	–	–	1.04	0.87
		After MVR	549	14.4%	14	37	18	26.2%	0.99	0.80
		∆ MVR-BY	190	5.0%	1	22	–	–	− 0.05 (− 4.8%)	− 0.07 (− 7.7%)
	I_10_	Base year	714	10.1%	13	16	–	–	1.04	0.87
		After MVR	1066	15.1%	14	36	31	38.8%	0.95	0.77
		∆ MVR-BY	352	5.0%	2	20	–	–	− 0.09 (− 8.9%)	− 0.10 (− 11.4%)
	*I*_11_	Base year	930	10.1%	13	15	–	–	1.04	0.87
		After MVR	1390	15.1%	15	39	41	45.6%	0.92	0.75
		∆ MVR-BY	460	5.0%	3	24	–	–	− 0.12 (− 11.4%)	− 0.12 (− 14.0%)
	*I*_12_	Base year	1203	10.3%	13	16	–	–	1.04	0.87
		After MVR	1786	15.3%	14	22	51	53.4%	0.91	0.74
		∆ MVR-BY	583	5.0%	1	6	–	–	− 0.13 (− 12.7%)	− 0.13 (− 14.8%)
*Rectum resection*	*I*_1_	Base year	25	5.1%	14	11	–	–	1.01	0.87
		After MVR	75	15.0%	14	45	3	20.5%	1.00	0.86
		∆ MVR-BY	49	9.9%	1	34	–	–	− 0.01 (− 1.4%)	− 0.01 (− 1.5%)
	*I*_2_	Base year	148	9.6%	14	19	–	–	1.01	0.87
		After MVR	302	19.6%	16	62	9	32.9%	0.96	0.84
		∆ MVR-BY	154	10.0%	2	43	–	–	− 0.05 (− 4.8%)	− 0.03 (− 3.6%)
	*I*_3_	Base year	276	8.9%	14	16	–	–	1.01	0.87
		After MVR	585	18.9%	17	48	17	50.1%	0.92	0.81
		∆ MVR-BY	309	10.0%	3	32	–	–	− 0.10 (− 9.6%)	− 0.06 (− 6.5%)
	*I*_4_	Base year	464	10.2%	14	17	–	–	1.01	0.87
		After MVR	920	20.2%	18	46	29	62.9%	0.86	0.78
		∆ MVR-BY	456	10.0%	4	29	–	–	− 0.15 (− 14.8%)	− 0.08 (− 9.7%)
	*I*_5_	Base year	850	12.4%	14	18	–	–	1.01	0.87
		After MVR	1534	22.4%	20	49	44	78.3%	0.82	0.74
		∆ MVR-BY	684	10.0%	7	31	–	–	− 0.20 (− 19.4%)	− 0.13 (− 14.6%)
	*I*_6_	Base year	1169	13.5%	14	18	–	–	1.01	0.87
		After MVR	2035	23.5%	21	46	56	89.3%	0.78	0.72
		∆ MVR-BY	866	10.0%	7	28	–	–	− 0.24 (− 23.3%)	− 0.15 (− 17.5%)
	*I*_7_	Base year	25	5.1%	14	11	–	–	1.01	0.87
		After MVR	50	10.1%	14	39	4	14.6%	1.00	0.86
		∆ MVR-BY	25	5.0%	0	29	–	–	− 0.01 (− 1.3%)	− 0.01 (− 1.5%)
	*I*_8_	Base year	148	9.6%	14	19	–	–	1.01	0.87
		After MVR	225	14.6%	15	48	8	26.2%	0.97	0.84
		∆ MVR-BY	77	5.0%	1	29	–	–	− 0.05 (− 4.6%)	− 0.03 (− 3.8%)
	*I*_9_	Base year	276	8.9%	14	16	–	–	1.01	0.87
		After MVR	430	13.9%	16	43	15	35.8%	0.92	0.81
		∆ MVR-BY	155	5.0%	3	27	–	–	− 0.09 (− 9.1%)	− 0.06 (− 6.8%)
	*I*_10_	Base year	464	10.2%	14	17	–	–	1.01	0.87
		After MVR	692	15.2%	17	41	24	49.5%	0.87	0.78
		∆ MVR-BY	228	5.0%	3	24	–	–	− 0.14 (− 14.0%)	− 0.09 (− 10.2%)
	*I*_11_	Base year	850	12.4%	14	18	–	–	1.01	0.87
		After MVR	1192	17.4%	19	43	34	61.0%	0.83	0.73
		∆ MVR-BY	342	5.0%	5	25	–	–	− 0.18 (− 17.6%)	− 0.14 (− 15.5%)
	*I*_12_	Base year	1169	13.5%	14	18	–	–	1.01	0.87
		After MVR	1602	18.5%	20	42	42	70.3%	0.81	0.71
		∆ MVR-BY	433	5.0%	6	24	–	–	− 0.21 (− 20.5%)	− 0.16 (− 18.8%)
*Radical prostatectomy*	*I*_1_	Base year	162	17.4%	12	15	–	–	1.12	1.09
		After MVR	255	27.4%	13	41	10	24.5%	1.06	1.00
		∆ MVR-BY	93	10.0%	1	25	–	–	− 0.05 (− 4.8%)	− 0.09 (− 7.8%)
	*I*_2_	Base year	230	16.5%	12	17	–	–	1.12	1.09
		After MVR	369	26.5%	13	43	13	34.5%	1.04	0.95
		∆ MVR-BY	139	10.0%	2	25	–	–	− 0.08 (− 7.1%)	− 0.13 (− 12.3%)
	*I*_3_	Base year	385	15.9%	12	14	–	–	1.12	1.09
		After MVR	627	25.9%	14	38	22	43.4%	1.01	0.90
		∆ MVR-BY	242	10.0%	3	24	–	–	− 0.11 (− 10.1%)	− 0.19 (− 17.2%)
	*I*_4_	Base year	485	15.4%	12	13	–	–	1.12	1.09
		After MVR	800	25.4%	15	34	28	48.9%	0.99	0.89
		∆ MVR-BY	315	10.0%	3	21	–	–	− 0.13 (− 11.7%)	− 0.20 (− 18.0%)
	*I*_5_	Base year	–	–	–	–	–	–	–	–
		After MVR	–	–	–	–	–	–	–	–
		∆ MVR-BY	–	–	–	–	–	–	–	–
	*I*_6_	Base year	571	16.1%	12	13	–	–	1.12	1.09
		After MVR	1281	36.1%	16	43	38	58.1%	0.96	0.87
		∆ MVR-BY	711	20.0%	5	29	–	–	− 0.16 (− 13.9%)	− 0.21 (− 19.7%)
	*I*_7_	Base year	730	16.7%	12	13	–	–	1.12	1.09
		After MVR	1607	36.7%	17	39	48	56.9%	0.96	0.85
		∆ MVR-BY	877	20.0%	5	26	–	–	− 0.16 (− 14.3%)	− 0.23 (− 21.6%)
	*I*_8_	Base year	807	15.6%	12	12	–	–	1.12	1.09
		After MVR	1845	35.6%	18	39	61	60.1%	0.98	0.84
		∆ MVR-BY	1038	20.0%	6	27	–	–	− 0.14 (− 12.2%)	− 0.25 (− 22.8%)
	*I*_9_	Base year	807	15.6%	12	12	–	–	1.12	1.09
		After MVR	2364	45.5%	20	48	77	72.6%	0.96	0.84
		∆ MVR-BY	1557	30.0%	8	36	–	–	− 0.16 (− 13.9%)	− 0.25 (− 23.1%)
	*I*_10_	Base year	571	16.1%	12	13	–	–	1.12	1.09
		After MVR	1281	36.0%	16	41	36	49.6%	0.97	0.87
		∆ MVR-BY	710	20.0%	4	28	–	–	− 0.15 (− 13.1%)	− 0.22 (− 20.3%)
	*I*_11_	Base year	730	16.7%	12	13	–	–	1.12	1.09
		After MVR	1606	36.6%	17	41	44	50.1%	0.97	0.85
		∆ MVR-BY	876	20.0%	5	28	–	–	− 0.14 (− 12.9%)	− 0.24 (− 22.3%)
	*I*_12_	Base year	807	15.6%	12	12	–	–	1.12	1.09
		After MVR	1845	35.6%	18	39	55	49.1%	1.00	0.83
		∆ MVR-BY	1038	20.0%	6	27	–	–	− 0.12 (− 10.6%)	− 0.26 (− 23.5%)
*Total knee arthroplasty*	*I*_1_	Base year	109	9.1%	15	36	–	–	1.01	1.10
		After MVR	173	14.4%	15	57	5	8.0%	1.00	1.09
		∆ MVR-BY	64	5.3%	0	21	–	–	− 0.01 (− 0.9%)	− 0.01 (− 1.3%)
	*I*_2_	Base year	500	9.5%	15	19	–	–	1.01	1.10
		After MVR	1022	19.5%	17	70	24	26.8%	0.97	1.07
		∆ MVR-BY	522	10.0%	2	51	–	–	− 0.04 (− 4.2%)	− 0.03 (− 2.8%)
	*I*_3_	Base year	1078	9.9%	15	15	–	–	1.01	1.10
		After MVR	2168	19.9%	16	29	45	49.4%	0.91	0.93
		∆ MVR-BY	1090	10.0%	1	13	–	–	− 0.10 (− 10.2%)	− 0.17 (− 15.7%)
	*I*_4_	Base year	1974	11.5%	15	14	–	–	1.01	1.10
		After MVR	3694	21.5%	16	25	75	69.6%	0.86	0.89
		∆ MVR-BY	1720	10.0%	1	11	–	–	− 0.16 (− 15.4%)	− 0.21 (− 19.1%)
	*I*_5_	Base year	3063	11.6%	15	13	–	–	1.01	1.10
		After MVR	5697	21.6%	17	23	117	76.0%	0.81	0.77
		∆ MVR-BY	2634	10.0%	2	10	–	–	− 0.20 (− 20.1%)	− 0.33 (− 29.9%)
	*I*_6_	Base year	4293	12.7%	15	15	–	–	1.01	1.10
		After MVR	7666	22.7%	17	23	144	74.8%	0.79	0.71
		∆ MVR-BY	3373	10.0%	2	8	–	–	− 0.22 (− 22.1%)	− 0.40 (− 35.8%)
	*I*_7_	Base year	109	9.1%	15	36	–	–	1.01	1.10
		After MVR	186	15.6%	15	61	5	7.6%	1.00	1.09
		∆ MVR-BY	77	6.5%	0	24	–	–	− 0.01 (− 0.9%)	− 0.01 (− 1.3%)
	*I*_8_	Base year	494	9.4%	15	18	–	–	1.01	1.10
		After MVR	1018	19.4%	16	37	21	22.9%	0.97	1.07
		∆ MVR-BY	524	10.0%	1	19	–	–	− 0.04 (− 4.1%)	− 0.03 (− 2.8%)
	*I*_9_	Base year	1078	9.9%	15	15	–	–	1.01	1.10
		After MVR	2168	19.9%	16	29	45	47.8%	0.91	0.93
		∆ MVR-BY	1090	10.0%	1	13	–	–	− 0.10 (− 10.2%)	− 0.17 (− 15.7%)
	*I*_10_	Base year	1974	11.5%	15	14	–	–	1.01	1.10
		After MVR	3694	21.5%	16	25	71	60.9%	0.86	0.89
		∆ MVR-BY	1720	10.0%	1	11	–	–	− 0.15 (− 14.8%)	− 0.21 (− 19.5%)
	*I*_11_	Base year	3063	11.6%	15	13	–	–	1.01	1.10
		After MVR	5697	21.6%	17	22	101	59.9%	0.82	0.76
		∆ MVR-BY	2634	10.0%	2	9	–	–	− 0.19 (− 18.7%)	− 0.34 (− 30.7%)
	*I*_12_	Base year	4293	12.7%	15	15	–	–	1.01	1.10
		After MVR	7666	22.7%	17	23	133	64.8%	0.81	0.70
		∆ MVR-BY	3373	10.0%	2	8	–	–	− 0.20 (− 20.2%)	− 0.40 (− 36.7%)

Annotations: All absolute numbers are rounded to full values. All percentages are rounded to one-digit decimals. Absolute values for average patient travel time are shown in minutes. *I*_*n*_, iteration/calculation number *n*, BY means base year, *P*_r_ means all reassigned patients, *t* denotes the travel time threshold, *P* denotes all patients

Changes in the average outcome quality across all hospitals before and after strict MVR application represent the change in outcome quality attainable by strict MVR application and are used to calculate the number of avoidable complications in Table 1. The calculated average outcome quality was weighted by the case volume per hospital and was calculated using Microsoft Excel

Changes in the standard deviation of outcome quality across all hospitals before and after strict MVR application represent the level of uncertainty a patient incurs when randomly picking a hospital for treatment. In other words, the standard deviation represents the chance a patient has to actually receive the indicated average outcome quality across all hospitals as it describes the variation of outcome quality between different hospitals of the sample. Standard deviations were calculated using Microsoft Excel with the function STDEV.P

Data year: 2015

## Footnotes

1 CR and RR are separate treatment types of the procedure CRR (see Table 7 in the “Appendix”). As medical societies assign separate MVTs for the two treatment types [35], CR and RR are modeled separately.

2 The length of stay for RPE was inferred from DRGs M01A, M01B, and M04A using the G-DRG Report Browser 2015 to estimate the weighted average length of stay.

## Code availability

The code is available upon well-reasoned request.
